# Ketamine ameliorates depressive-like behaviors in mice through increasing glucose uptake regulated by the ERK/GLUT3 signaling pathway

**DOI:** 10.1038/s41598-021-97758-7

**Published:** 2021-09-13

**Authors:** Xin Ouyang, Zhengjia Wang, Mei Luo, Maozhou Wang, Xing Liu, Jiaxin Chen, JianGuo Feng, Jing Jia, Xiaobin Wang

**Affiliations:** 1 Department of Anesthesiology, The Affiliated Hospital of Southwest Medical University, No. 25, Taiping Road, Luzhou, 646000 Sichuan Province People’s Republic of China; 2 Heart Center, Beijing Anzhen Hospital, Captial Medical University, Beijing, 100020 People’s Republic of China; 3 Department of Anesthesiology, The Second Affiliated Hospital of Chengdu Medical College, China National Nuclear Corporation 416 Hospital, Chengdu, Sichuan People’s Republic of China

**Keywords:** Neuroscience, Medical research

## Abstract

To investigate the effects of ketamine on glucose uptake and glucose transporter (GLUT) expression in depressive-like mice. After HA1800 cells were treated with ketamine, 2-[N-(7-Nitrobenz-2-oxa-1,3-diazol-4-yl)Amino]-2-Deoxyglucose (2-NBDG) was added to the cells to test the effects of ketamine on glucose uptake, production of lactate, and expression levels of GLUT, ERK1/2, AKT, and AMPK. Adult female C57BL/6 mice were subjected to chronic unpredictable mild stress (CUMS), 27 CUMS mice were randomly divided into the depression, ketamine (i.p.10 mg/kg), and FR180204 (ERK1/2 inhibitor, i.p.100 mg/kg) + ketamine group. Three mice randomly selected from each group were injected with ^18^F-FDG at 6 h after treatment. The brain tissue was collected at 6 h after treatment for p-ERK1/2 and GLUTs. Treatment with ketamine significantly increased glucose uptake, extracellular lactic-acid content, expression levels of GLUT3 and p-ERK in astrocytes and glucose uptake in the prefrontal cortex (*P* < 0.05), and the immobility time was significantly shortened in depressive-like mice (*P* < 0.01). An ERK1/2 inhibitor significantly inhibited ketamine-induced increases in the glucose uptake in depressive-like mice (*P* < 0.05), as well as prolonged the immobility time (*P* < 0.01). The expression levels of p-ERK1/2 and GLUT3 in depressive-like mice were significantly lower than those in normal control mice (*P* < 0.01). Ketamine treatment in depressive-like mice significantly increased the expression levels of p-ERK1/2 and GLUT3 in the prefrontal cortex (*P* < 0.01), whereas an ERK1/2 inhibitor significantly inhibited ketamine-induced increases (*P* < 0.01).Our present findings demonstrate that ketamine mitigated depressive-like behaviors in female mice by activating the ERK/GLUT3 signal pathway, which further increased glucose uptake in the prefrontal cortex.

## Introduction

Traditional antidepressants have limited efficacies, obvious side effects, and delayed response time of weeks to months^1^. Ketamine, as an N-methyl-d-aspartate (NMDA) receptor antagonist, produces rapid (within hours) antidepressant actions in patients who are resistant to typical antidepressants^2,3^. Notably, ketamine also produces schizophrenia-like positive and negative symptoms in healthy human subjects^4^. Accordingly, such adverse effects limit the clinical application of ketamine as an antidepressant. Therefore, better understanding of the molecular mechanisms underlying the rapid antidepressant effects of ketamine is of great significance for the development of new therapeutic targets.

Accumulating evidence suggests that glucose metabolism in the brain is decreased in depressed patients and in animal models of depression^5,6^. Furthermore, ketamine has been shown to significantly increase glucose uptake in relevant brain areas^7,8^. However, the mechanism by which ketamine promotes glucose metabolism in the brain has remained unclear.

The extracellular-regulated kinase (ERK) signaling pathway, mediated through NMDA receptors, plays an important role in several cellular activities^9,10^. Studies suggest that activation and expression of ERK is reduced in the prefrontal cortices of post-mortem brains from depressed patients who committed suicide^11^. Ketamine has also been reported to ameliorate severe traumatic-event-induced antidepressant-resistant depression in a rat model through the activation of ERK in the prefrontal cortex^12^, which subsequently regulates intracellular glucose metabolism by pyruvate kinase subtype M2 (PKM2)^13^. Glucose transporters (GLUTs) regulate glucose transport in brain cells, which in turn affects brain function and energy metabolism^14,15^. Thus, we hypothesized that ketamine may produce antidepressant effects via regulating glucose metabolism in the prefrontal cortex by increasing ERK phosphorylation levels, which would further upregulate the expression of GLUTs.

Astrocytes, as local communication elements with multiple functions^16^, are the most abundant cell type in the mammalian brain^17^. Astrocytes that are surrounded by capillaries in the brain express high levels of GLUTs^18^. In the present study, we used cultured astrocytes and established a mouse model of depression to investigate the effects of ketamine on glucose uptake and the expression of ERK/GLUTs in astrocytes within the prefrontal cortices of depressed mice.

## Results

### Ketamine increases 2-NBDG uptake, production of lactate, and protein levels of GLUT3 and P-ERK1/2

**Figure 1:**
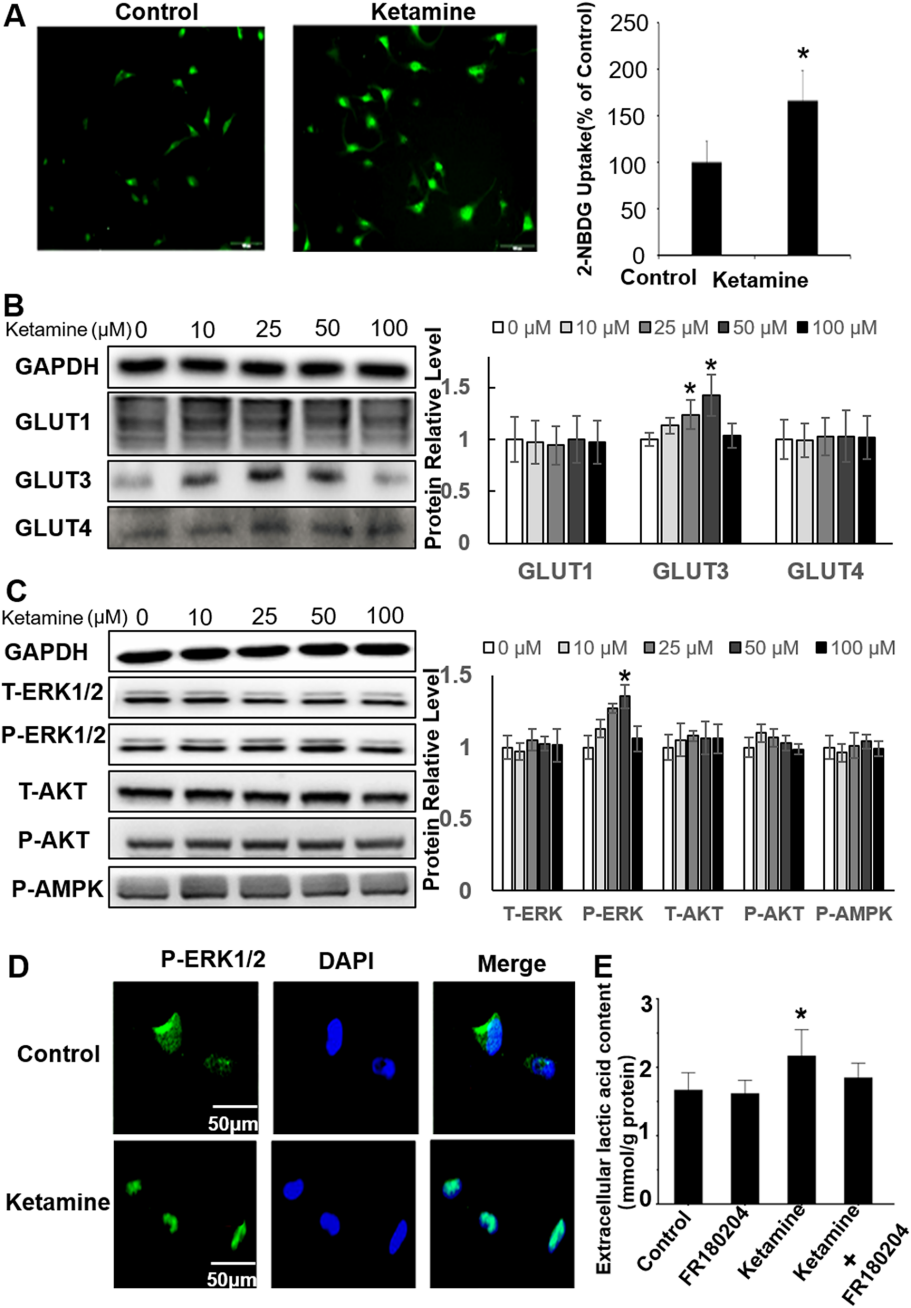
Ketamine increases the 2-NBDG uptake, protein levels of GLUT3 and P-ERK1/2, and production of lactate in HA1800 cells. (**A**) The intracellular fluorescence of 2-NBDG was examined by confocal microscopy. (**B**) The expression of GLUT1, GLUT3 and GLUT4 were examined by Western blots at 6 h post ketamine treatment with different concentration (0, 10, 25, 50 and 100 μM); (**C**) Western blots analysis was used to test the levels of P-ERK1/2, P-AKT and P-AMPK at 6 h post ketamine treatment with different concentration (0, 10, 25, 50 and 100 μM); (**D**) The subcellular location of P-ERK1/2 response to ketamine treatment was detected by immunofluorescent staining; (**E**) The production of lactate was tested by a lactate detection kit. Vs control, *P < 0.05 and **P < 0.01. Immunoblotting signals were quantified via Image J software.

Compared with that of the control group, treatment with 50 μM/L of ketamine for 6 h significantly increased the uptake of 2-NBDG (*P* < 0.01, Fig. 1A). Ketamine also dramatically enhanced the protein levels of GLUT3 (*P* < 0.05, Fig. 1B) and P-ERK1/2 (*P* < 0.01, Fig. 1C). Immunofluorescent staining further showed the increased nuclear distributions of P-ERK1/2 after ketamine treatment for 6 h when compared with that of the control (Fig. 1D). No significant differences were found in P-AKT or P-AMPK levels in the ketamine group relative to those in the control group (*P* > 0.05, Fig. 1C).

Additionally, we found significantly increased production of lactate following treatment with 50 μM/L of ketamine relative to that of the control group (control vs. ketamine: 1.67 ± 0.25 mM/g protein vs 2.17 ± 0.38 mM/g protein, *P* < 0.05, Fig. 1E), while no significance was found when compared with that following treatment with ketamine and an ERK1/2 inhibitor (FR180204; 1.85 ± 0.21 mM/g protein, *P* > 0.05, Fig. 1E).

### Ketamine ameliorates depressive-like behaviors in mice via the ERK1/2 signaling pathway

**Figure 2:**
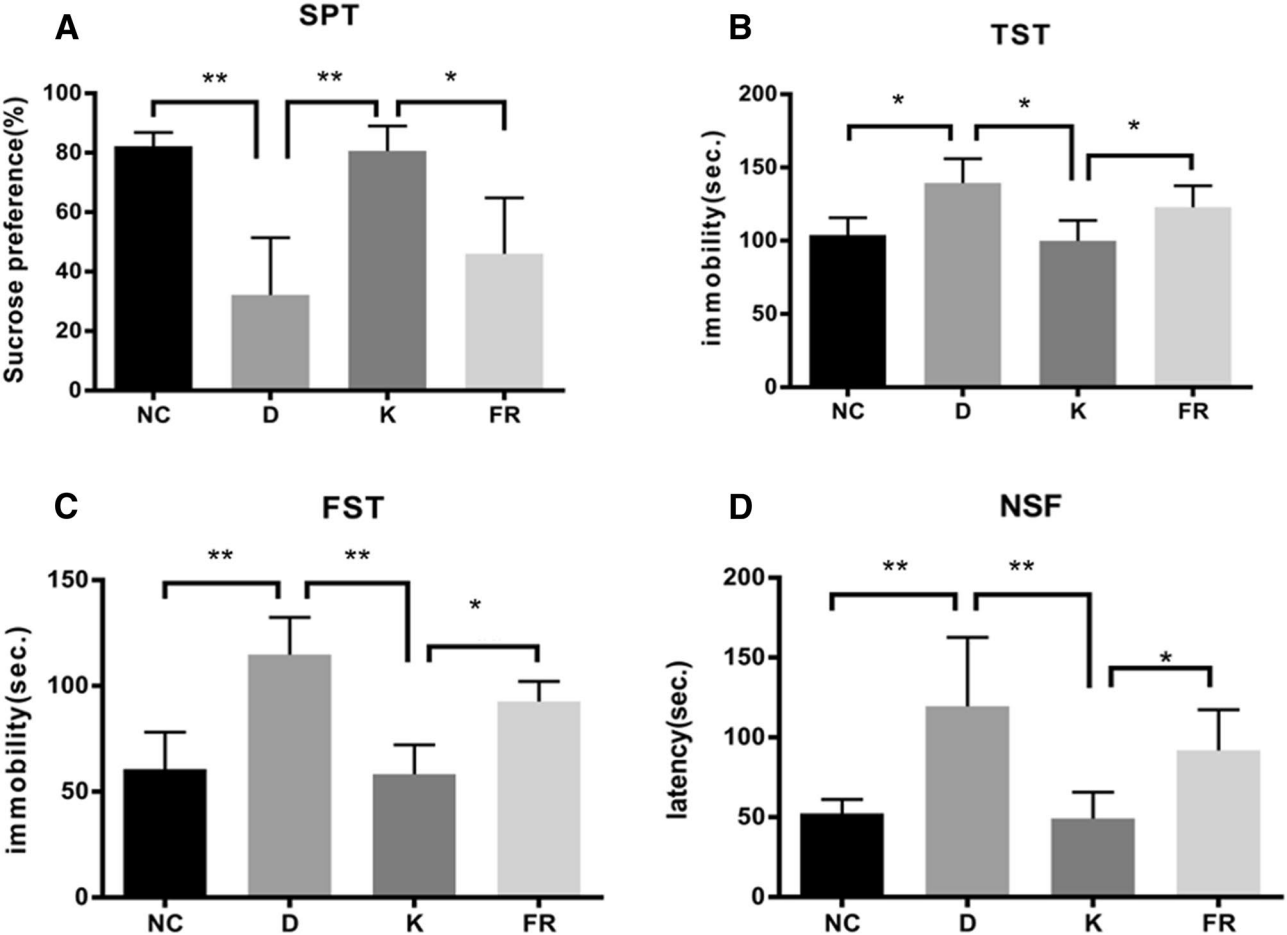
Effects of ketamine on depressive-like behaviors in CUMS mice. (**A**) The percentage of the consumption of sucrose-sweetened water in the SPT. (**B**) The immobility time in the TST. (**C**) The immobility time in the FST. (**D**) The latency to feed in the NSF test. *NC* normal control group, *D* depression group, *K* ketamine group, *FR* ERK1/2 inhibitor (FR180204) group; n = 6. *P < 0.05, **P < 0.01.

Results in the SPT (Fig. 2A) revealed an overall significantly reduced preference for sucrose in depressed mice when compared to that of control mice (depressed vs. control: 32.25 ± 17.56% vs. 82.35 ± 4.06%, *P* < 0.01). Ketamine treatment dramatically increased the percentage of sucrose-sweetened water consumption in depressive-like mice (80.66 ± 7.65%, *P* < 0.01). However, this effect of ketamine on daily sucrose intake was reversed by an ERK1/2 inhibitor (46.03 ± 17.20%, *P* < 0.05).

In the TST (Fig. 2B), depressive-like mice exhibited significantly longer immobility times compared with those of normal controls (depressed vs. control: 139.33 ± 15.22 s vs 103.83 ± 10.95 s, *P* < 0.05). Ketamine treatment significantly decreased the immobility time of depressed mice (99.83 ± 12.85 s, *P* < 0.05), which was partially reversed by additional treatment with an ERK1/2 inhibitor (122.83 ± 13.50 s, *P* < 0.05).

In the FST (Fig. 2C), a significant increase in immobility time was found in depressive-like mice compared to that of control mice (depressed vs control: 114.83 ± 16.03 s vs. 60.67 ± 16.02 s, *P* < 0.01). The immobility times of depressive-like mice treated with ketamine were significantly reduced when compared with those of depressive-like mice treated with saline (58.33 ± 12.67 s, *P* < 0.01). In contrast, addition of an ERK1/2 inhibitor increased the immobility times of depressive-like mice treated with ketamine compared with those of depressive-like mice only treated with ketamine (92.67 ± 8.65 s, *P* < 0.05).

In the NSF test (Fig. 2D), depressive-like mice presented a significant increase in the latency to feed compared to that of the control group (depressed vs. control: 108.06 ± 39.42 s vs. 46.02 ± 8.12 s, *P* < 0.01). Ketamine treatment significantly decreased the latency to feed of depressive-like mice to 44.32 ± 15.21 s (*P* < 0.01), which was increased in depressive-like mice when they were additionally treated with an ERK1/2 inhibitor combined with ketamine (82.04 ± 23.30 s, *P* < 0.05).

### Ketamine increases glucose uptake in the prefrontal cortices of depressive-like mice via the ERK1/2 signaling pathway

**Figure 3:**
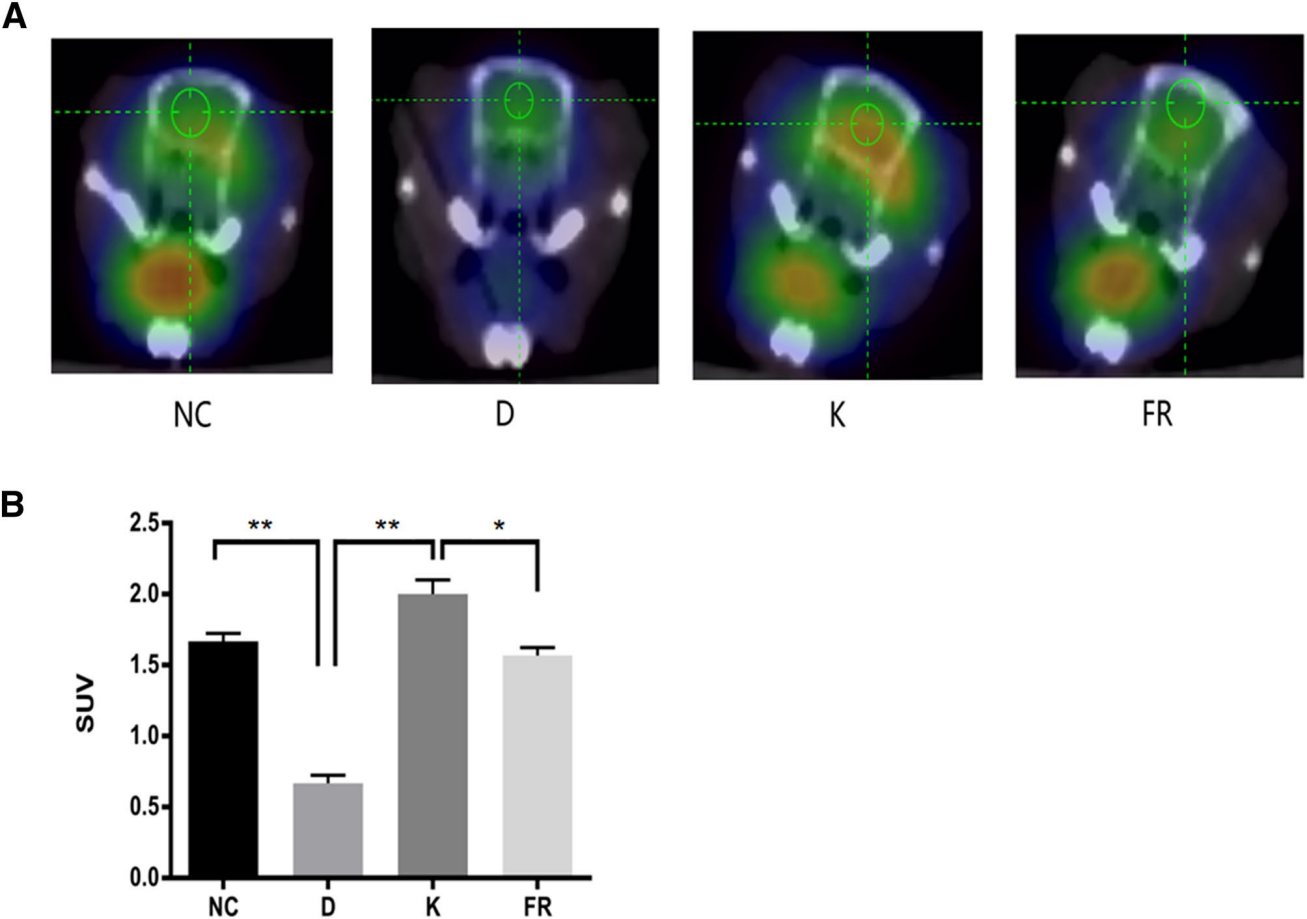
Ketamine increases glucose uptake in the prefrontal cortices of depressive-like mice. (**A**) ^18^F-FDG PET CT brain imaging; the more red, the more glucose uptake. (**B**) The SUV was prefrontal-cortex SUV (normalized to that of the whole-brain SUV). *NC* normal control group, *D* depression group, *K* ketamine group, *FR* ERK1/2 inhibitor (FR180204) group; n = 3; *P < 0.05, **P < 0.01. Images were analyzed using ASI Pro VM software (Siemens Medical Solutions, USA).

As shown in Fig. 3, compared with that of the control group, the decrease of glucose uptake was found in the prefrontal cortices of depressive-like mice, as demonstrated by significantly lower normalized SUV values (*P* < 0.01). Ketamine treatment significantly increased glucose uptake, as demonstrated by higher normalized SUV values in depressive-like mice compared with those of depressive-like mice without ketamine treatment (*P* < 0.01). Inhibition of ERK1/2 by FR180204 in ketamine-treated depressive-like mice decreased glucose uptake, as demonstrated by a significant decrease in normalized SUV values (*P* < 0.05).

### Ketamine upregulates the protein levels of P-ERK1/2 and GLUT3 in the prefrontal cortices of depressed mice via the ERK1/2 signaling pathway

**Figure 4:**
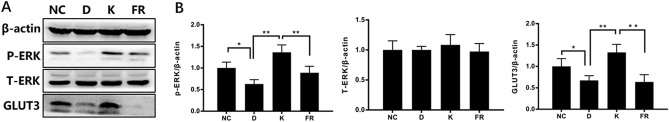
Ketamine upregulates the protein levels of P-ERK1/2 and GLUT3 in the prefrontal cortices. (**A**) Western-blot detection of related protein expression. (**B**) Relative expression levels of P-ERK1/2, T-ERK1/2, and GLUT3; *NC* normal control group, *D* depression group, *K* ketamine group, FR: ERK1/2 inhibitor (FR180204) group; n = 6; *P < 0.05, **P < 0.01. Protein levels of P-ERK1/2, T-ERK1/2, and GLUT3 in the prefrontal cortices were quantified by Image J software.

As shown in Fig. 4, the protein levels of P-ERK1/2 (*P* < 0.05) and GLUT3 (*P* < 0.05) in the prefrontal cortex were decreased in depressed mice compared with those in control mice. Ketamine significantly increased the expression of P-ERK1/2 (*P* < 0.01) and GLUT3 (*P* < 0.01) in the prefrontal cortex of depressed mice. Treatment with an ERK1/2 inhibitor (FR180204) decreased these protein levels in the prefrontal cortices of depressed mice treated with ketamine (*P* < 0.01).

### Astrocytes, but not neurons, are involved in the effects of ketamine mitigating depressive-like behaviors in mice via the ERK1/2 signaling pathway

**Figure 5:**
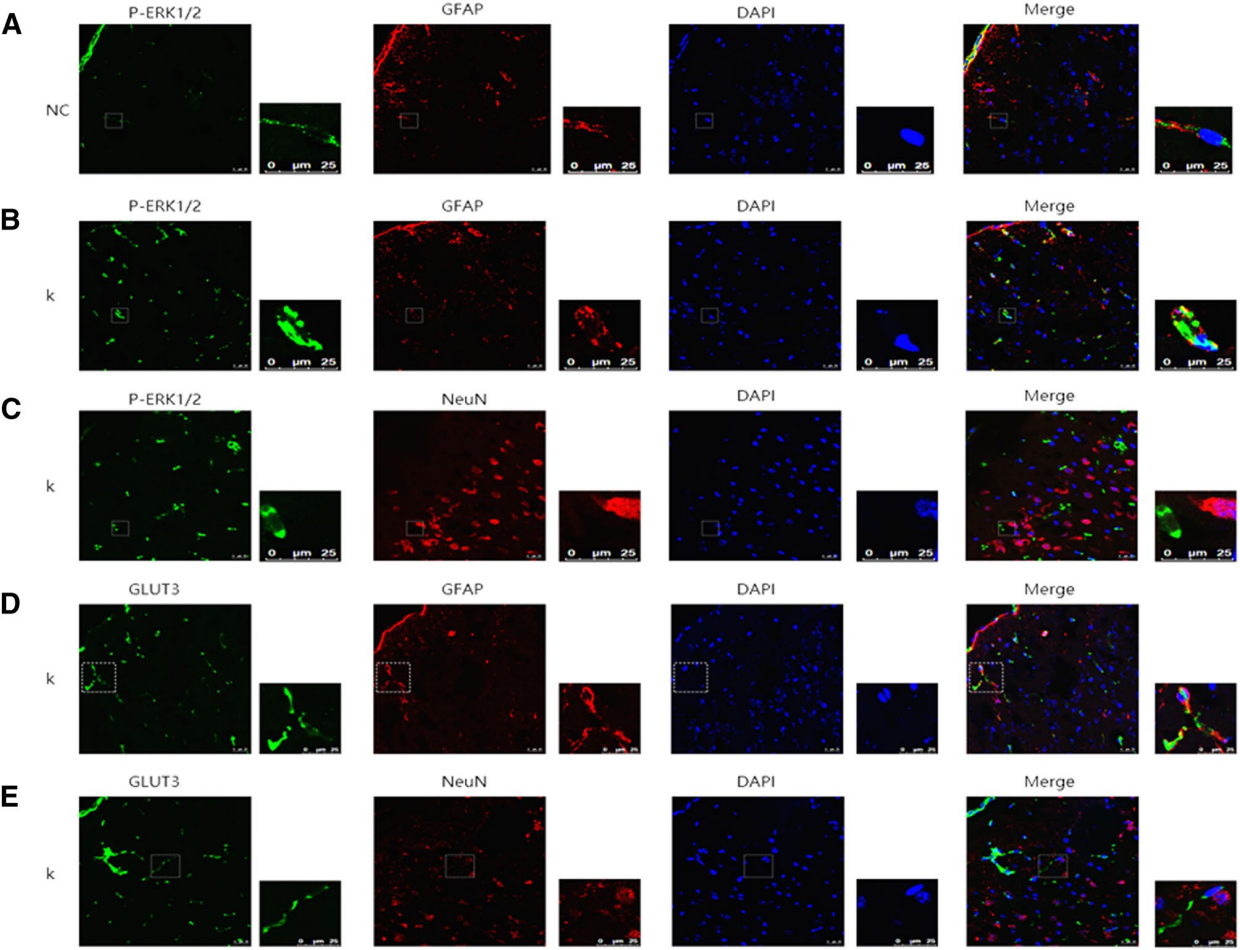
Immunofluorescence micrographs in the prefrontal cortices of depressive-like mice (**A**,**B**): Stained with the P-ERK1/2 antibodies (green) and GFAP antibodies (red). (**C**): Stained with the P-ERK1/2 antibodies (green) and neuron antibodies (red). (**D**): Stained with the P-ERK1/2 antibodies (green) and GLUT3 antibodies (red). (**E**): Stained with the P-ERK1/2 antibodies (green) and GLUT3 antibodies (red). *NC* normal control group, *K* ketamine group, *GFAP* astrocyte marker, *NeuN* neuron marker; scale bar = 25 μM.

We also measured the expression levels and spatial distributions of P-ERK1/2 and GLUT3 in the prefrontal cortices of mice. As shown in Fig. 5, upregulation of P-ERK1/2 and GLUT3 in the prefrontal cortices of mice in the ketamine group was mainly distributed in astrocytes; the upregulation of P-ERK1/2 and GLUT3 in the prefrontal cortex in the ketamine group was less distributed in neurons. Furthermore, P-ERK1/2 in the prefrontal cortices of mice in the ketamine group was aggregated in the nuclei of astrocytes.

## Discussion

Although several studies have reported that ketamine exerts rapid and sustained antidepressant effects in patients with depression, the partial underlying mechanisms have been elucidated^19–25^. Additionally, ketamine-induced antidepressant effects are accompanied by an increase in the cerebral metabolic rate of glucose (CMRGlc)^26^. Therefore, it is possible that ketamine-enhanced CMRGlc is due to an increase in glucose uptake within the brain. Astrocytes play numerous complex functions in the central nervous system^17^, and a reduction in astrocytes and their related markers are associated with the pathology of major depressive disorder^27,28^. Hence, in the present study, we assessed the effects of glucose uptake induced by ketamine in human-derived astrocytes (HA1800). Our results indicated that treatment with 50 μM (equivalent to subanesthetic dose^29^) of ketamine for 6 h remarkably enhanced glucose uptake in astrocytes. However, the mechanism of glucose uptake induced by ketamine in astrocytes remains unclear.

Relevant studies have shown that GLUTs play a key role in the glucose uptake of astrocytes^30^, among which GLUT1, GLUT3, and GLUT4 play important roles^31^. Hence, in the current study, we detected the expression levels of GLUT1, GLUT3, and GLUT4 induced by ketamine in astrocytes. Our findings indicated that ketamine induced glucose absorption in astrocytes through GLUT3 instead of GLUT1 or GLUT4. Under physiological conditions, the expression levels of GLUT1 and GLUT3 are detected at relatively low levels, and astrocytes exhibit increased expression levels of GLUT3 under some stress conditions, such as mild ischemia^32^. Additionally, the endotoxin lipopolysaccharide (LPS)^33^ can lead to an increase in glucose uptake. GLUT4 has been identified as an insulin-responsive transporter, and its regulatory mechanisms differ from those of GLUT1 or GLUT3^34^. Since GLUT3 transports extracellular glucose into cells at a rate of approximately seven-fold that of GLUT1^35^, we hypothesize that ketamine-induced increases in GLUT3 levels in HA1800 cells in our present study may lead to the significant increase in glucose uptake. The results of the present study are consistent with those of Tomioka et al. in which they reported the effects of ketamine on glucose uptake by GLUT3 expressed in Xenopus oocytes^36^. In addition, Iasevoli et al. found that ketamine increases GLUT3 mRNA expression in rats^37^. However, it is still unclear which signaling pathway may be associated with ketamine-induced increases in GLUT3 levels in astrocytes.

Several studies have shown that active ERK1/2, AKT, and AMPK signaling pathways accelerate glucose uptake in astrocytes^38,39^. The findings of our present study indicated that ketamine treatment markedly increased the expression levels of p-ERK1/2, while it did not influence the expression levels of p-AKT or p-AMPK. Furthermore, immunostaining assays showed that p-ERK1/2 translocated into the nucleus following ketamine treatment, which may generate physiological actions such as proliferation and differentiation^40,41^. However, whether p-ERK1/2 further regulates the expression of GLUT3 after entering the nucleus requires further investigation.

**Figure 6:**
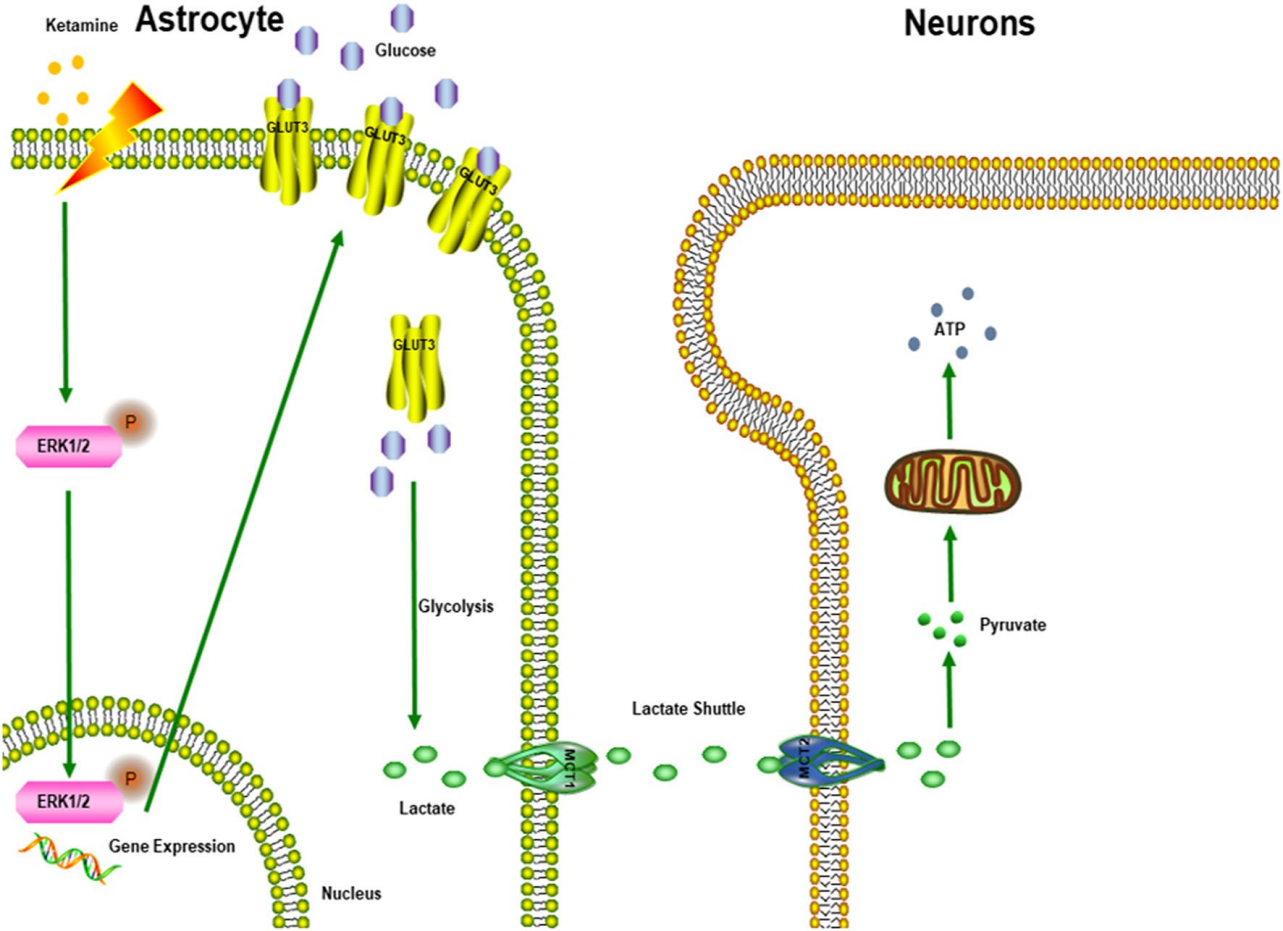
The ANLS (Astrocyte-neuron lactate shuttle) model.

Our present findings revealed that ketamine increased glucose uptake in astrocytes. However, we did not explore which metabolic process participated in this glucose uptake. An ANLS model (Fig. 6) showed that glucose is taken up by astrocytes located around blood vessels and is converted to lactate, the latter of which is transferred into neurons as energy substrates^42^. Although we demonstrated an increased lactate level in astrocytes, whether lactate provides energy to neurons requires further investigation.

On the basis of the above results in vitro, we further explored the related phenomena and mechanisms by establishing a mouse model of depression. CUMS, which induces long-lasting depressive-like behavior for up to several months, is a well-established model of depression in rodents and can be reversed by chronic treatments with traditional antidepressant agents^43–45^. Compared to those of males, females have higher incidence rates of major depressive disorder^46,47^. Experimental studies have also reported a sex difference in depressive-like behaviors, with more remarkable depressive-like behaviors in female rather male LPS-exposed or CUMS mice^48,49^. Therefore, we established a mouse model of depression in female mice in the current study. When we designed the experiment, we deliberately chose the stimulation factors that would not cause damage to the limbs or affect the motor ability of mice, all kinds of stress involved in the experiment were mild and non-violent as we previously reported^5^, therefore, in the study, we did not again detect the influences of the treatment administered on the motor ability of mice before performing the OFT. Male rats have been previously reported to exhibit depressive-like behavior after 4–8 weeks of CUMS^50–52^. In the present study, we successfully established depressed-like female mice after 21 days of CUMS, as demonstrated by results of the SPT and FST. Therefore, this provides a model to explore potential targets for the treatment of depression.

In the current study, results of behavioral tests in depressed-like mice indicated that ketamine treatment dramatically ameliorated depressive-like behaviors. This finding is consistent with previous experiments performed by Berman et al.^53^ and Zarate et al.^54^. In addition, many authors report behavioral effects in mice submitted to the CUMS or corticosterone model with ketamine at 3 or 5 mg/kg. We use ketamine at 10 mg/kg, which do not cause schizophrenia, because the subclinical doses of ketamine (10 mg/kg) used in the study of antidepressant effect of ketamine in mice has been recorded in the literature^55^.

Early studies have reported a relationship of ketamine with phosphorylation levels of the ERK signaling pathway and anti-depressant effects^23,56^. In our present study, we found that inhibition of ERK signaling significantly eliminated the improved effects of ketamine on depressive-like behaviors of depressed mice, further confirming the essential roles of ERK signaling in the anti-depressant effects of ketamine.

The prefrontal cortex, considered as a large part of the neural system crucial for normal socio-emotional and executive function in humans and other primates, has been suggested to be tightly associated with depression^57,58^. Animal studies have documented a dramatic decrease in glucose uptake within the prefrontal cortex by ^18^F-FDG micro-PET/CT, which is increased after anti-depressive treatment^59^. A Clinical PET study reported lower glucose metabolism in the prefrontal cortices of depressed patients at rest, as compared with that of control subjects^6^. Another randomized controlled study also found that low-dose ketamine increased glucose uptake in the prefrontal cortex in treatment-resistant depressed patients^7^. Together with these previous studies, our present results further demonstrated an association of antidepressant effects of ketamine with glucose metabolism in the prefrontal cortex. Both astrocytes and neurons are located in the prefrontal cortex. However, it has remained unclear whether astrocytes and/or neurons participate in the antidepressant effects of ketamine on glucose metabolism in the prefrontal cortex. Furthermore, few studies have elucidated the mechanisms of ketamine on glucose uptake involving the ERK/GLUT3 signaling pathway in the prefrontal cortex. In the current study, we found that the protein levels of GLUT3 and P-ERK1/2 were significantly decreased in the prefrontal cortices of depressed mice compared to those in control mice. Low-dose ketamine dramatically increased these protein levels in the prefrontal cortex. Such antidepressant effects of ketamine were subsequently inhibited by the additional treatment of an ERK1/2 inhibitor. Our results are consistent with previous findings by Lee et al.^12^ and Iasevoli et al.^37^. Immunofluorescent staining further confirmed that astrocytes, but not neurons, exhibited enhanced expression levels of GLUT3 and P-ERK1/2 in the prefrontal cortex. Previous studies have revealed that nuclear translocation of P-ERK1/2 plays an important role in the initiation of proliferation and differentiation^40,41^. Furthermore, Watanabe et al.^60^ reported that GLUT3 expression in cancer cells by DNA-damaging agents was dependent on the ERK pathway. Therefore, in our present study, we hypothesize that nuclear localization of P-ERK1/2 in astrocytes subsequently enhanced glucose metabolism in the prefrontal cortex via promoting the expression of GLUT3. However, the potential mechanisms of P-ERK1/2 regulating the expression of GLUT3, the involvement of glucose uptake in astrocytes, and energy metabolism in peripheral neurons remain unclear. Hence, further studies are warranted to elucidate these mechanisms.

In conclusion, our present study revealed that ketamine ameliorated depressive-like behaviors in mice by enhancing glucose uptake in the prefrontal cortex via activating the ERK/GLUT3 signaling pathway.

## Materials and methods

### Ethics statement

This research strictly followed the principles of animal use in the China Laboratory Animal Science Association. The study obtained the approval of the Animal Ethics Committee of Affilated Hosptal of Southwest Medical University (Approval#: 20180306038), and all experiments were performed in accordance with relevant guidelines and regulations. In order to reduce animal suffering, all surgery was performed under anesthesia with pentobarbital sodium. Here we state that all methods in this study are reported in accordance with ARRIVE guidelines.

### Cell culture

The normal human astrocyte cell line, HA1800, was purchased from Boster Biological Technology, Ltd. (Wuhan, China). HA1800 cells were cultured in DMEM supplemented with 10% fetal bovine serum (FBS), penicillin (100 U/mL), and streptomycin (100 µg/mL) in a 5% CO_2_ atmosphere at 37 °C.

### Glucose uptake assays

Glucose uptake was evaluated by 2-(N-(7-Nitrobenz-2-oxa-1,3-diazol-4-yl)Amino)-2-Deoxyglucose (2-NBDG) (APExBIO Technology LLC, Houston, TX, USA, ID B6035). HA1800 cells were cultured in 24-well plates. After reaching 60% confluence, the cells were treated with 0, 10, 25, 50, 100 μmol/L ketamine(Gutian Pharmaceutical Company, ID1504152) or 10 μmol/L FR180204 (ERK1/2 inhibitor:Beyotime Institute of Biotechnology, Shanghai, China, ID SD5978-25 mg) for 6 h. After treatment, 100 µM of 2-NBDG was added to the cells, and the cells were then incubated for 20 min at 37 °C. After washing three times with cold phosphate-buffered saline (PBS) to terminate uptake, the cells were fixed with 4% paraformaldehyde (PFA) for 20 min and were then washed three times with cold PBS. Furthermore, the fluorescent intensity of each culture labeled with 2-NBDG was measured by a fluorescent microscope (Bio-Tek Instruments Inc., Winooski, VT, USA).

### Western blotting

In the present study, HA1800 cells, were seeded in 10-cm dishes and kept for 24 h at 37 °C. After exposure to 10 µM of ketamine or an ERK1/2 inhibitor, cells were washed with PBS and lysed with radio-immunoprecipitation assay (RIPA) lysis buffer supplemented with a protease-inhibitor and phosphatase-inhibitor all-in-one cocktail (Beyotime Institute of Biotechnology, Shanghai, China). Protein concentrations were quantified by an Enhanced BCA Protein Assay Kit (Beyotime Institute of Biotechnology, Shanghai, China, ID0010S). The total protein contents of samples were subjected to 10% sodium-dodecyl-sulfate polyacrylamide gel electrophoresis (SDS-PAGE) and were then electro-transferred for 1 h to nitrocellulose-filter (NC) membranes. The membranes were blocked with 5% nonfat milk in TBST buffer (20 mM Tris–Cl pH 8.0, 0.05%Tween-20, 150 uM NaCl). Then, the membranes were incubated at 4 °C overnight with appropriate primary antibodies), including the following: anti‐GLUT1(Beyotime Institute of Biotechnology, Shanghai, China, ID AF1015), anti‐GLUT3 (Santa Cruz, ID sc-74497), anti‐GLUT4 (Santa Cruz, ID sc-53566), anti‐phospho-AMPK (Beyotime Institute of Biotechnology, Shanghai, China, ID AF2677), anti‐phospho-AKT (Cell Signaling, ID #9271), anti‐phospho-ERK (Beyotime Institute of Biotechnology, Shanghai, China, ID AM071), anti‐total-ERK (Santa Cruz, ID sc-135900), anti‐GAPDH (Proteintech, ID 60004-1-lg), and anti‐β‐ACTIN (Proteintech, ID 66009-1-lg) . Subsequently, the membranes were incubated with species-matched secondary antibodies, as follows: goat-anti-rabbit IgG for anti‐GLUT1, anti‐phospho-AMPK, anti‐phospho-AKT, anti‐phospho-ERK, and anti‐total-ERK; and goat-anti-mouse IgG for anti‐GLUT3, anti‐GLUT4, anti‐GAPDH, and anti‐β‐ACTIN. Specific reactive proteins were detected by enhanced chemiluminescence (ECM). Signals were developed by Fusion Solo (Vilber Lourmat, Collégien, France). Immunoblotting signals were quantified via Image J software (Version 1.47v, https://imagej.nih.gov/ij/).

### Immunostaining analysis

Immunostaining analysis was carried out to detect localization of total-ERK1/2 and phospho-ERK1/2 in astrocytes. HA1800 cells were seeded in 24-well plates overnight. After treatment with ketamine, cells were immediately fixed with 4% PFA for 20 min and were then washed three times with cold PBS. After blocking with 1% bovine serum albumin (BSA) for 1 h at room temperature, the cells were incubated with specific primary antibodies at 4 °C overnight. After washing with PBS, the cells were incubated with fluorescein isothiocyanate (FITC)-conjugated goat anti-rabbit IgG (Bioss, ID bs-0295G-FITC, dilution, 1:500) for 1 h at room temperature. Next, cellular nuclei were stained with 4′,6-diamidino-2-phenylindole (DAPI). After addition of antifade mounting medium, fluorescent signals were immediately examined under a fluorescent microscope. The fluorescent signals were quantified via Image J software 1.47v (National Institutes of Health, USA).

### Lactate production assays

HA1800 cells were seeded in 10-cm dishes and maintained for 24 h, after which they were treated with ketamine and/or an ERK1/2 inhibitor for 6 h. Thereafter, the culture medium from each sample was transferred to an Eppendorf (EP) tube, and lactate production was then measured by a lactate detection kit (Nanjing Jiancheng Bioengineering Institute, Nanjing, China) according to the manufacturer’s protocol.

### Animals

**Figure 7:**
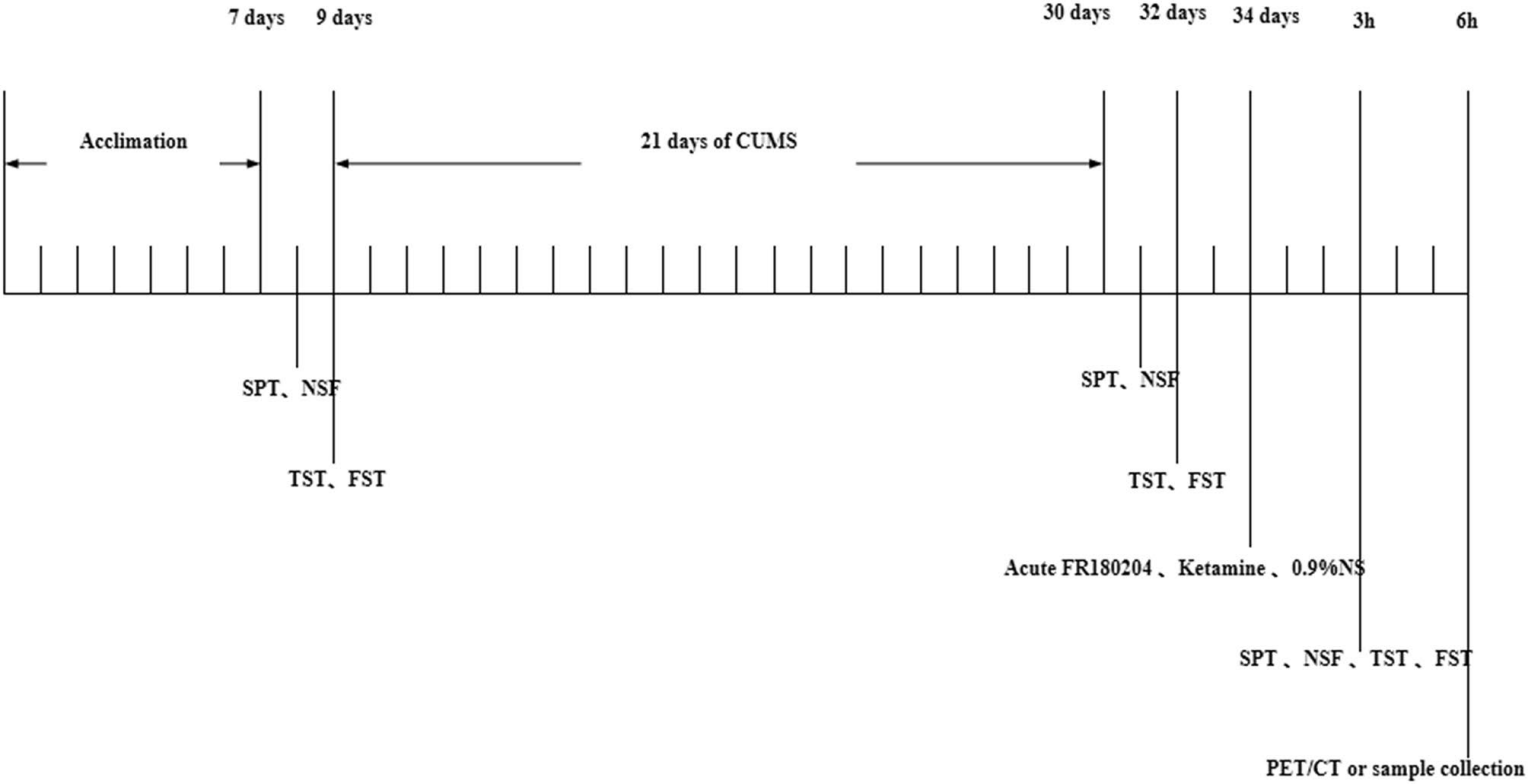
Flowchart of experimental setup.

Female C57BL/6 mice (18–22 g, 6–8 weeks, n = 90) were obtained from Chengdu Dossy Experimental Animals Company of China, and were maintained under a controlled temperature (22 °C ± 2 °C) and humidity (50% ± 20%) with a 12/12-h light/dark cycle (lights on at 7:00 a.m.). All animals were provided food and water ad libitum, except when they were 24-h food-deprived prior to the novelty suppressed feeding (NSF) test and 24-h water-deprived prior to the sucrose preference test (SPT). Mice were acclimatized for 7 days prior to the start of experiments. The experimental flowchart is presented in Fig. 7. After baseline assessment of depressive-like behaviors (excluding 9 mice with abnormal baseline data), 81 mice were then randomly assigned to the following two groups: (1) normal control group (NC, n = 9) without any treatment; and (2) test group (n = 72) with chronic unpredictable mild stress (CUMS). Successful establishment of our CUMS model was determined by significant differences in depressive-like behaviors acquired on experimental day 21 when compared with those of the control group. Ultimately, 27 CUMS mice were randomly assigned to the following three groups: (1) depressive-like group (D group, n = 9), administered intraperitoneally (i.p.) with 0.9% saline; (2) ketamine group (K group, n = 9), administered i.p. with 10 mg/kg of ketamine (this dose is appropriate for producing anti-depressive effects in rats, and the rats were still conscious for flowing injection of this dose^61^); and (3) ERK1/2 inhibitor (FR180204) group (FR group, n = 9), administered i.p. with 100 mg/kg of FR180204 followed by 10 mg/kg of ketamine, i.p. at 1 h later. Ketamine (Gutian Pharmaceutical Company, Fujian, China) was dissolved in saline (0.9%), the ERK1/2 inhibitor, FR180204 (Beyotime Institute of Biotechnology, Shanghai, China) was dissolved in methyl cellulose (0.1%, Beyotime Institute of Biotechnology, Shanghai, China). Depressive-like behaviors (n = 6 for each group) and ^18^F-FDG micro positron emission tomography (PET)/computed tomography (CT) imaging (n = 3 for each group) were assessed followed by ketamine treatments at 3 h and 6 h, respectively.

### Chronic unpredictable mild stress (CUMS)

CUMS, as described by Wang et al.^62^, was performed with some modifications. The experimental stressors were as follows: horizontal oscillation for 1 h; physical restraint for 1 h; tail clipping for 5 min; wetting litter for 12 h; cage tilting for 12 h; swimming in 10 °C water for 3 min; swimming in 45 °C water for 3 min; continuous illumination during the dark phase; and water or food deprivation. Mice were administered these stressors in a random order for 21 days, such that we avoided using the same stress for two consecutive days, and a given stress was not used more than two times in a week.

### Sucrose preference test (SPT)

The SPT was carried out on experimental day 8, 31, and 34 in each mouses home cage, as previously reported^12^. At the beginning of the experiment, mice were trained to habituate to sucrose solution by placing two bottles of either 2.5% sucrose solution or tap water in each cage for 24 h, with the positions of these bottles changing every 12 h. All mice were then water-deprived for 24 h prior to the SPT. On day 3, each mice was given free access to same two bottles of either 2.5% sucrose solution or tap water for 1 h, with the positions of these bottles changing every 30 min. The sucrose preference rate (%) was calculated as follows: sucrose consumption mL/(total water + sucrose consumption ml) × 100%.

### Novelty suppressed feeding (NSF) test

The NSF test was performed on experimental days 8, 31, and 34, as previously described^63^. The latency of each mouse to approach and eat food was measured in a novel environment following an extended period (up to 24 h) of food deprivation. Approximately 24 h after the removal of the food, mice were placed in an illuminated and sound-proofed box (40 × 40 × 40 cm) with a small piece of mouse chow placed in the center of the box. Each mouse was placed in the corner of the testing arena, and the time until the first feeding episode was recorded within 5 min.

### Tail suspension test (TST)

The tail suspension test (TST) was performed on experimental days 9, 32, and 34, according to a previous protocol^64^. Mice were individually suspended by their tails using adhesive tape placed approximately 1 cm from the tip of the tail, keeping mice positioned 15 cm above the tabletop. The entire procedure lasted for 6 min, and the immobility duration was recorded for the last 4 min. The mice were considered immobile only when they hung down passively and were completely motionless.

### Forced swimming test (FST)

The FST was performed on experimental days 9, 32, and 34, as described by Dulawa et al.^65^. The mice were forced to swim in an open cylindrical container (diameter of 10 cm and height of 30 cm) filled with 20 cm of water at 23–25 °C. The total immobility, swimming, and climbing times were recorded during the last 4 min of a 6-min test using a video camera positioned directly above the cylinder. A mouse was judged as immobile when it floated without struggling and only maintained its head above the water.

### ^18^F-FDG micro-PET/CT

Prior to PET scans, mice were fasted for 12 h. Each mouse was injected with approximately 14.5–16.6 MBq of ^18^F FDG via the tail vein. After an uptake period of 40 min in a quiet and dimly-lit environment, each mouse was anaesthetized with 1% sodium pentobarbital (50 mg/kg, Beijing Chemical Reagent Company, Beijing, China), and was then placed in the prone position for imaging. PET signals and CT scans were acquired for 10 min in a micro-PET/CT scanner (Siemens, Germany). The raw PET data were binned into a single frame, and multiple planar reconstruction (MPR) images were obtained by the MAP reconstruction method. ASI Pro VM software ((https://www.siemens-healthineers.com/en-us/, Siemens Medical Solutions, USA) was used for analysis and semi-quantification of the brain images in the region of interest (ROI), yielding the standardized uptake value (SUV) of the ROI that was representative of the metabolic rate in the corresponding regions. The prefrontal cortex was defined as the ROI. The prefrontal-cortex SUV was normalized to that of the whole-brain SUV within the same mouse, representing the level of glucose metabolism in the prefrontal cortex (normalized SUV values = prefrontal-cortex SUV/whole-brain SUV).

### Brain tissue collection

Mice were anesthetized by administration with 1% sodium pentobarbital (50 mg/kg) after behavioral tests. All mice were transcardially perfused with normal saline and were decapitated immediately. Whole brains were rapidly separated on ice. One side of the prefrontal cortex was quickly dissected and snap-frozen with liquid nitrogen prior to storage at − 80 °C until later use. The other side of the prefrontal cortex was promptly immersed in 4% paraformaldehyde overnight, and was then stored at − 80 °C for future use following dehydration in 20% and 30% sucrose solutions.

### Western blotting

Prefrontal cortices were mechanically homogenized in RIPA buffer combined with protease and phosphatase inhibitors (Solarbio, Beijing, China). Lysates were centrifuged (12,000 rpm for 15 min, at 4 °C), and total protein concentrations in the supernatants were quantified using BCA protein assay kits (Beyotime, Shanghai, China). The samples were separated by SDS-PAGE using a 10% gel, and the proteins were transferred to PVDF membranes (Millipore, Massachusetts, USA) using a semi-dry blotting apparatus (1.2 mA/cm^2^; 1.5 h). Subsequently, the membranes were blocked with 5% BSA in TBS (20 mM Tris–Cl, 150 mM NaCl, pH 8.0). The protein levels of GLUT3 (1:100; Santa Cruz, Dallas, Texas, USA), T-ERK1/2 (1:1000; Santa Cruz, Dallas, Texas, USA) P-ERK1/2 (1:1000; Beyotime, Shanghai, China), and β-actin (1:5000, loading control; Proteintech, Wuhan, Hubei, China) were determined via overnight incubation in corresponding primary antibodies diluted in TBS-T (20 mM Tris–Cl, 150 mM NaCl, 0.05% Tween-20, pH 8.0). The membranes were then incubated with horseradish-peroxidase-conjugated secondary antibodies (anti-mouse antibody, 1:5000, Proteintech, Wuhan, Hubei, China; or anti-rabbit antibody, 1:2500, Proteintech, Wuhan, Hubei, China) for 60 min. The immunoreactive bands were developed using a chemiluminescence kit (Beyotime, Shanghai, China). All blocking and incubation steps were followed by three washes (5 min each wash) of the membranes with TBS-T. The optical densities (ODs) of the protein blots were quantified using the Image J software (National Institutes of Health, USA). The phosphorylation levels of ERK1/2 were determined as a ratio of the OD of the phosphorylated protein band to the OD of the total protein band. GLUT3 protein levels were determined as a ratio of the OD of the GLUT3 band to the OD of the β-actin band.

### Immunofluorescent assays

The prefrontal cortices of mice were analyzed via immunofluorescent assays. Coronal sections (5-μm thickness) were prepared and immersed in 4% PFA for 30 min, followed by three washes in TBST. After blocking non-specific binding via 1.5% BSA for 1 h, sections were then incubated with corresponding primary antibodies overnight at 4 °C, as follows: GFAP (1:50; Proteintech, Wuhan, Hubei, China) and P-ERK1/2 (1:100; Beyotime, Shanghai, China); GFAP and GLUT3 (1:100; Santa Cruz, Dallas, Texas, USA); NeuN (1:50; Proteintech, Wuhan, Hubei, China); P-ERK1/2; and GFAP and T-ERK1/2 (1:100; Santa Cruz, Dallas, Texas, USA). After washing three times with TBST, the secondary antibody mix with FITC-conjugated goat anti-mouse IgG (1:200; Bioss, Beijing, China) and Cy3-conjugated goat anti-rabbit IgG (1:200; Bioss, Beijing, China) was added to cover the tissue and was incubated at room temperature for 1 h in the dark. Sections were then incubated with DAPI at room temperature for 5 min. Fluorescent images were obtained, and image analysis was applied to quantify immunoreactive signals.

### Statistical analysis

Statistical analysis was carried out using the GraphPad Prism software 8.0.2 (https://www.graphpad.com/). The data are presented as the mean ± standard error of the mean (SEM). Differences among experimental groups were determined by one-way analysis of variance (ANOVA) followed by Bonferroni tests for post-hoc comparisons. *P* < 0.05 was considered statistically significant.

## ARRIVE Guidelines Statement

Here we state that all methods in this study are reported in accordance with ARRIVE guidelines.

## Supplementary Information

Supplementary Information.
