# Supplementary material for: Ketamine ameliorates depressive-like behaviors in mice through increasing glucose uptake regulated by the ERK/GLUT3 signaling pathway
Source: Sci Rep. 2021 Sep 13;11:18181. doi: 10.1038/s41598-021-97758-7 (PMC8437933; doi:10.1038/s41598-021-97758-7)
Supplement: Supplementary file 1 — Supplementary Information. [file 41598_2021_97758_MOESM1_ESM.pdf]

Supplementary Materials

1. Figure 2. Replicates of blots

Figure 2B

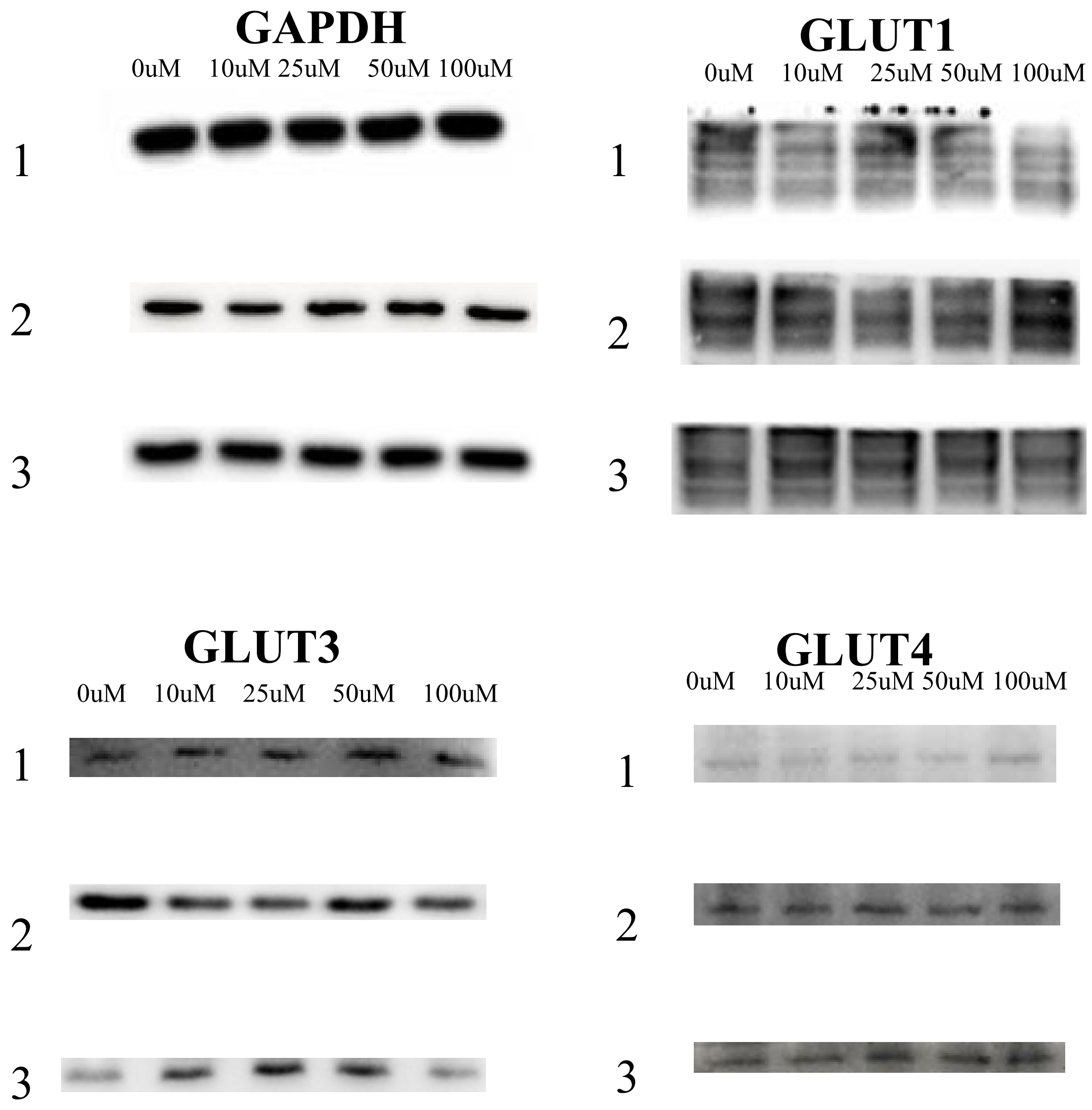

Figure 2C

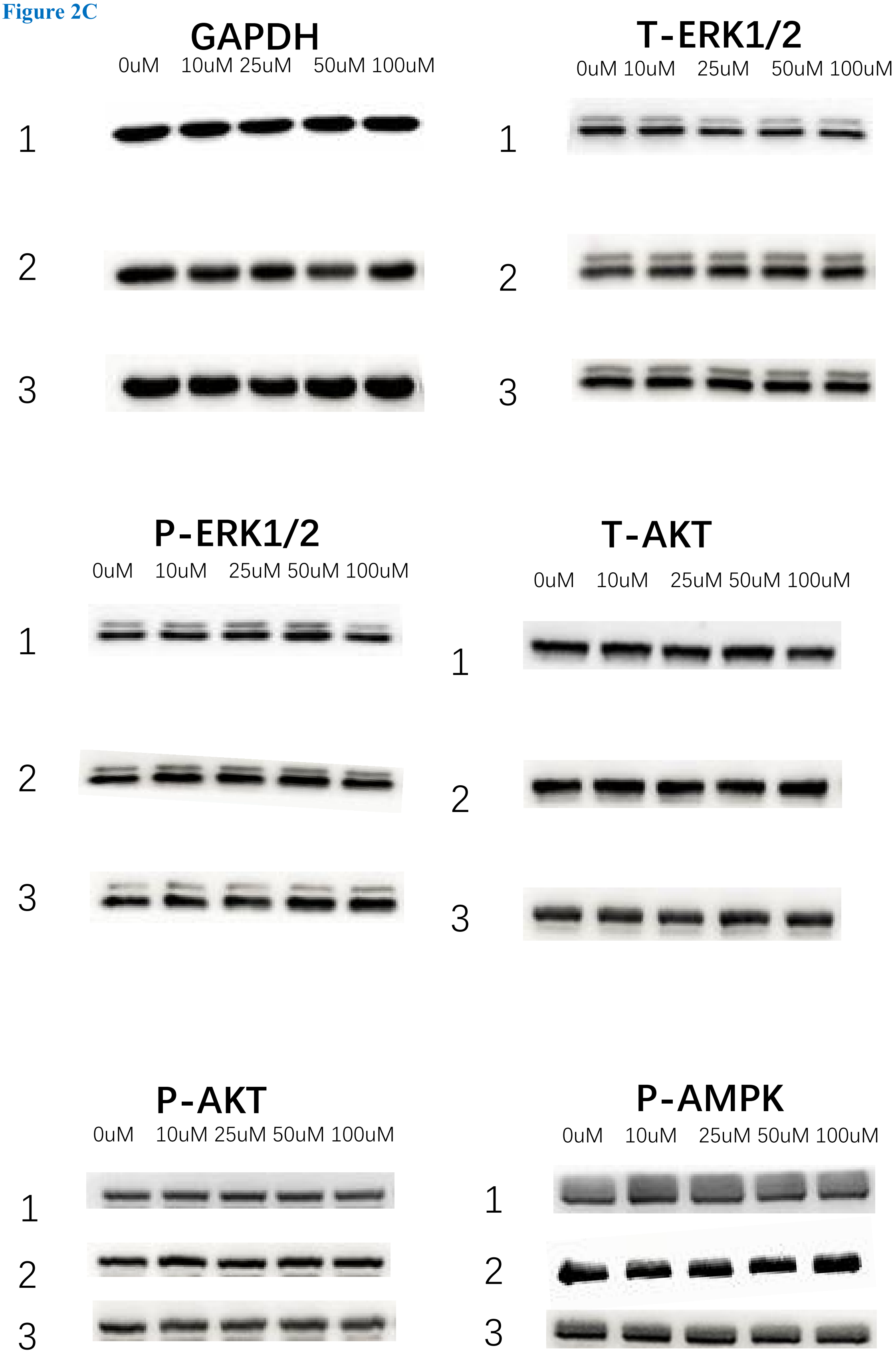

GAPDH
0uM 10uM 25uM 50uM 100uM
1
2
3
T-ERK1/2
0uM 10uM 25uM 50uM 100uM
1
2
3
P-ERK1/2
0uM 10uM 25uM 50uM 100uM
1
2
3
T-AKT
0uM 10uM 25uM 50uM 100uM
1
2
3
P-AKT
0uM 10uM 25uM 50uM 100uM
1
2
3
P-AMPK
0uM 10uM 25uM 50uM 100uM
1
2
3

Figure 5A.

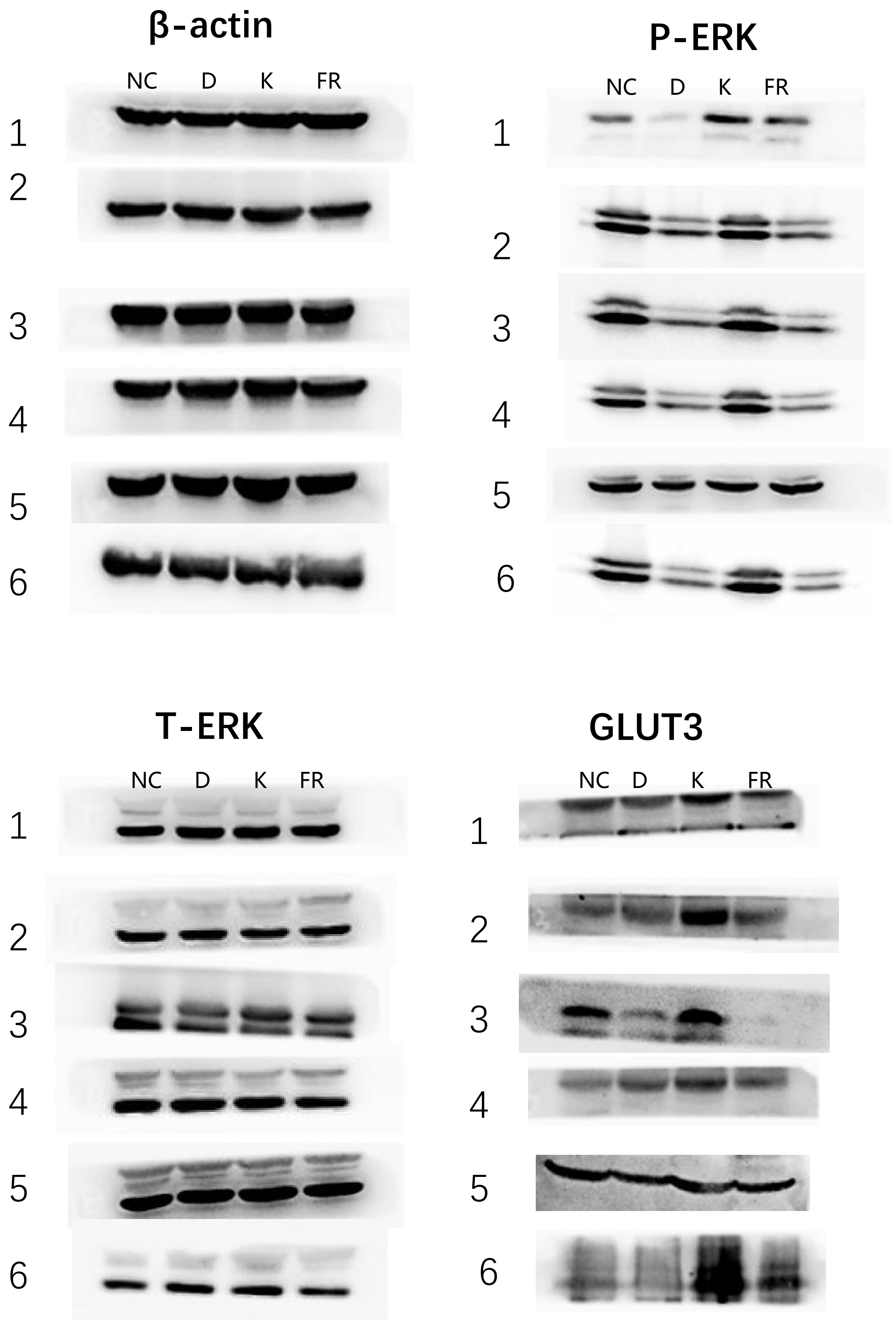
β-actin
NC D K FR
1
2
3
4
5
6
P-ERK
NC D K FR
1
2
3
4
5
6
T-ERK
NC D K FR
1
2
3
4
5
6
GLUT3
NC D K FR
1
2
3
4
5
6

Certificate of English Language Editing

Certificate of English Language Editing

Manuscript Title:

Ketamine ameliorates depressive-kike behaviors in mice through increasing glucose uptake regulated by the ERK/GLUT3 signaling pathway

Date of Revision:

January 8, 2021

Abstract:

Objectives The aim of the present study was to investigate the effects of ketamine on glucose uptake and glucose transporter (GLUT) expression in astrocytes within the prefrontal cortices of depressed mice.
Methods The cultured normal human astrocytic cell line, HA1800, and female C57BL/6 depressed mice were used for the following experiments. After HA1800 cells were treated with 50 μM of ketamine for 6 h, 2-[N-(7-Nitrobenz-2-oxa-1,3-diazol-4-yl)Amino]-2-Deoxyglucose (2-NBDG) was added to the cells to test the effects of ketamine on glucose uptake, production of lactate, and expression levels of GLUT, ERK1/2, AKT, and AMPK. Adult female C57BL/6 mice were subjected to chronic unpredictable mild stress (CUMS) to establish a murine model of depression. Normal female C57BL/6 mice were used as the control group (n=9), and 27 CUMS mice were randomly divided into the depression group (n=9), ketamine group (n=9), and ERK1/2 inhibitor + ketamine group (n=9); mice in the above CUMS groups were intraperitoneally injected with saline, ketamine (10 mg/kg), and an ERK1/2 inhibitor (FR180204) (100 mg/kg) + ketamine (10 mg/kg), respectively. Three mice were randomly selected from each group and were injected with 18F-FDG through the tail vein. Then, micro positron emission tomography (PET)/computed tomography (CT) was used to observe changes in glucose uptake in the prefrontal cortex of each mouse. The rest of the mice in each group were evaluated for depressive-like behaviors at 3 h...

This document certifies that the manuscript listed above was copy edited for proper English language at LetPub. All of our language editors are native English speakers with long-term experience in editing scientific and technical manuscripts. We are committed to leveling the playing field for researchers whose native language is not English.

- Neither the research content nor the authors' intended meaning were altered in any way during the editing process.
- Documents receiving this certification should be considered ready for publication where language issues are concerned. *However, the authors may accept or reject LetPub's suggestions and changes at their own discretion.*
- If you have any questions or concerns about this edited document, please contact us at support@letpub.com

LetPub
Your Manuscript Editor

LetPub is an author service brand owned and operated by Accdon LLC. Headquartered in the Boston area, we are a full-spectrum author services company with a large team of US-based certified language and scientific editors, ISO 17001 accredited translators, and professional scientific illustrators and animators. We advocate ethical publication practices and are an official member of the Committee on Publication Ethics (COPE).

For more information about our company, services, and partnership programs, please visit www.letpub.com.
© 2021 Accdon, LLC. All Rights Reserved. Tel: 1-781-202-9968 Email: info@accdon.com Address: 400 Fifth Ave, Suite 530, Waltham, MA 02451, United States
